# Nonnegotiable Symbolic Value and Sugar-Driven Food Habits in Indonesia: Mixed Methods Study Using a Digital Sociological Approach

**DOI:** 10.2196/77261

**Published:** 2026-02-27

**Authors:** Ewina Efriani Manik, Sudarsono Hardjosoekarto, Ricardi Adnan, Radhiatmoko Radhiatmoko, One Herwantoko, Hakiki Nurmajesty, Darwan Darwan, Farrah Eriska Putri, Astuti Sri Pawening

**Affiliations:** 1 Department of Sociology, Faculty of Social and Political Science, University of Indonesia, Pondok Cina, Depok, 16424, Indonesia, +62 21 7867222

**Keywords:** economic sociology, sugar market, symbolic value, uncontrollable consumption, digital research

## Abstract

**Background:**

The sugar market in Indonesia reflects the distinct consumer behavior shaped by economic and deeply rooted cultural factors. This study explores how symbolic values attached to sugar sustain persistent, often irrational or uncontrollable consumption, highlighting the need for a demand-side perspective in the economic sociology of sugar markets.

**Objective:**

This study analyzes the nonnegotiable symbolic value of sugar and its implication to uncontrollable consumption in Indonesia. Referring to the framework of product valuation in the social order of markets by Beckert, it offers insights into both the symbolic and material values of sugar.

**Methods:**

The applied method complements digital mixed method approaches used in prior research. Digital data from online news and YouTube were visualized through textual network analysis and social network analysis to describe the symbolic and material values of sugar. In-depth interviews with key actors and limited field observations on food and beverage labels were also conducted.

**Results:**

Findings reveal that the symbolic value of sugar increases significantly when processed into food or beverages, shaping food habits and habitus across diverse ethnic groups in Indonesia and reinforcing early dependence on sugar. Weak enforcement of labeling regulations on food and beverage packages further impedes shifts in consumer perceptions of the risks of excessive sugar consumption.

**Conclusions:**

This study contributes a demand-side perspective to the economic sociology of the sugar market, proposing strategies to address the sugar-driven food habits and habitus from the perspective of consumer behavior. Simultaneously, it assesses producer compliance with regulations on the sweetness level to reduce sugar consumption and the prevalence of noncommunicable diseases.

## Introduction

### Background

This study applies an economic sociology perspective to public health, particularly in controlling sugar consumption in Indonesia. In both traditional and modern contexts, sugar is not merely a sweetener for foods and beverages but also a cultural symbol of harmony, happiness, and social status, reproduced through food habits and habitus from an early age [1]. Economic growth, urbanization, lifestyle changes, and the promotion of sugar-based products have intensified sugar consumption, leading to adverse health outcomes. This prompts questions about the effect of uncontrolled sugar consumption on public health and the symbolic value of sugar on market demand in Indonesia.

The surge is particularly evident among Generation Z, for whom sweetened beverages (ie, bubble tea, milk coffee, and soft drinks) serve not merely to quench thirst but also to express pleasure, social identity, and self-expression as part of urban lifestyle. Sugar thus embodies a dual role: materially as a sweetener and symbolically as a powerful cultural element with a nonnegotiable symbolic value.

This symbolic value is reinforced through everyday practices and popular culture promoted by the media. Exchanging chocolates on Valentine’s Day or advertisements declaring “three Moo-Moo candies equal one glass of milk” and “there is a hint of sweetness to it” associate sweetness with affection and pleasure [2]. These messages sustain the position of sugar as a symbol of intimacy and happiness, fueling the demand for sugary and processed foods and beverages heavily promoted through print and television media [3].

From a public health perspective, excessive sugar consumption, aggravated by sedentary lifestyles, raises the risk of type 2 diabetes at a young age. Symptoms such as fatigue, excessive thirst, and slow-healing wounds often go unnoticed due to low public awareness of blood sugar monitoring [4]. Ironically, the cultural association of sweetness with enjoyment obscures these risks and burdens the national health care system.

From an economic sociology perspective, the sugar market is shaped not only by prices and regulations but also by social and cultural constructions of value. As Beckert [5] argues, product valuation in markets involves symbolic dimensions constructed through social and cognitive processes among market actors. While the World Health Organization (WHO) recommends limiting sugar to 25 grams per person (no more than 5 teaspoons) [6], Indonesia remains setting its national limit at 50 grams, with imports calculated on an assumption of 62 grams per person per day. This indicates a high dependency on sugar in both households and the food and beverage industry. Between 2020 and 2025, the World Bank discloses a 3.7% rise in sugar consumption in Indonesia, maintaining its position as the primary sugar importer since the 1980s [7].

Globally, such consumption patterns fuel noncommunicable diseases (NCDs), as observed in the United Kingdom. Since food consumption behavior is influenced by purchasing decisions, regulatory interventions should target not only nutritional and economic dimensions but also the symbolic and cultural factors shaping consumption patterns [8]. This study thus examines how the symbolic value of sugar influences market demand and public health in Indonesia through textual network analysis (TNA) and social network analysis (SNA).

### Objectives

This study explores 2 key aspects of the sugar market in Indonesia. First, it examines consumer behavior in reducing food habits and habitus shaped by sugar consumption. Second, it investigates producer compliance with regulations controlling sugar levels in foods and beverages.

To analyze the nonnegotiable symbolic value of sugar, this study adopted a network-based analytical approach integrating TNA and SNA. This approach is consistent with Hong and Lee [9], who used TNA to map long-term discourse structures and knowledge trends, and with Leem et al [10], who conceptualized TNA as a data-driven method for systematically uncovering conceptual structures and semantic relationships within text corpora. The application of SNA follows the framework proposed by Paterson et al [11], which emphasizes its role in identifying dominant actors, power relations, and policy dynamics within organizational networks.

The analysis was conducted in 5 stages. First, TNA was used to visualize the material value of sugar as reflected in discourses of standardization and regulation. Second, TNA captured the symbolic value of sugar by classifying patterns of cognitive anchoring and social positioning in public discourse. Third, SNA mapped dominant actors and network relations involved in policymaking on sweetness levels in packaged foods and beverages. Fourth, market observations were taken to assess compliance with sugar-content labeling. Fifth, all findings were synthesized to explain the persistence of sugar consumption, driven not only by material considerations or health information, but also by the socially and culturally institutionalized symbolic value of sugar.

Overall, this study addresses 3 main questions: First, how is the material value of sugar perceived, and how does it affect public health negatively? Second, how is the symbolic value of sugar socially and culturally constructed, leading to excessive consumption? Third, what sociological interventions can reduce sugar consumption while addressing its entrenched nonnegotiable symbolic value?

### Literature Review

#### The Construction of Symbolic and Material Value of Goods in Economic Sociology

Market sociology, as introduced by Beckert [5,12,13], mainly focuses on how product value and quality are created. Beckert [5] argues that “the more the value of products becomes detached from the fulfillment of purely functional needs, the more they depend upon symbolic assignments of value that must be constructed by market actors.” Consequently, understanding both symbolic and material qualities is important for meaningful market exchanges.

Several studies emphasize that material value remains relevant for predicting consumer preferences and brand choices. Similarly, other studies note that businesses enhance customer experiences by aligning products with consumer expectations [14] .

Quoting Durkheim, Beckert [15] posits that “value emerges from the symbolic connections made between goods and the socially rooted values.” Beckert further explains that “symbolic value is value from the symbolic meaning of objects.” It suggests that numerous goods exchanged in the market derive their primary value from symbolic meanings rather than physical qualities. Consumers consume products for their symbolic meanings rather than material use [14].

In this study, the symbolic value of sugar can be understood through diverse cultural contexts. Among Javanese communities, sweet foods signify harmony, gratitude, and positive social relations [1]. These values are reflected in everyday consumption practices and transmitted through food habitus from an early age. However, public health studies indicate that excessive sugar intake is a major risk factor for NCDs, including diabetes and obesity [16,17].

Beckert [5] presents the social order of markets framework, which theorizes that product valuation is socially and culturally patterned through 4 aspects: standardization, cognitive anchoring, normative legitimacy, and social positioning. Standardization appears in formal regulations such as the Regulation of the Ministry of Health (Permenkes) No. 30/2013 on daily sugar intake limits. Cognitive anchoring captures the cognitive and cultural associations between sweetness and togetherness in Javanese culinary traditions. Social positioning highlights the roles of key actors such as the sugar industry, government, and consumers in legitimizing and distributing the market value of sugar. Collectively, this framework enables a comprehensive analysis of the nonnegotiable symbolic value of sugar in Indonesia.

#### Sugar Market Valuation: The Nonnegotiable Symbolic Value

This study examines the nonnegotiable symbolic value in sugar consumption. Sugar plays a pivotal role in cultural communication as well as in economic and political contexts. Eating patterns are inseparable from cultural context, while food culture shapes individual identity [3].

Previous studies highlight the important symbolic value of food. Similarly, research linking socioeconomic status and consumption patterns discloses that food functions not only as nourishment but also as a bearer of social, cultural, and emotional meanings [18].

The nonnegotiable symbolic value of sugar appears in 2 stages. First, in material terms, low sugar consumption correlates with positive impacts on public health. On the other hand, high sugar consumption, as indicated by high sugar content (Brix value), leads to negative impacts on public health. Overcoming these impacts requires regulating sugar content in foods and beverages, imposing excise taxes on packaged foods and beverages, and promoting diabetes awareness.

**Figure 1.**
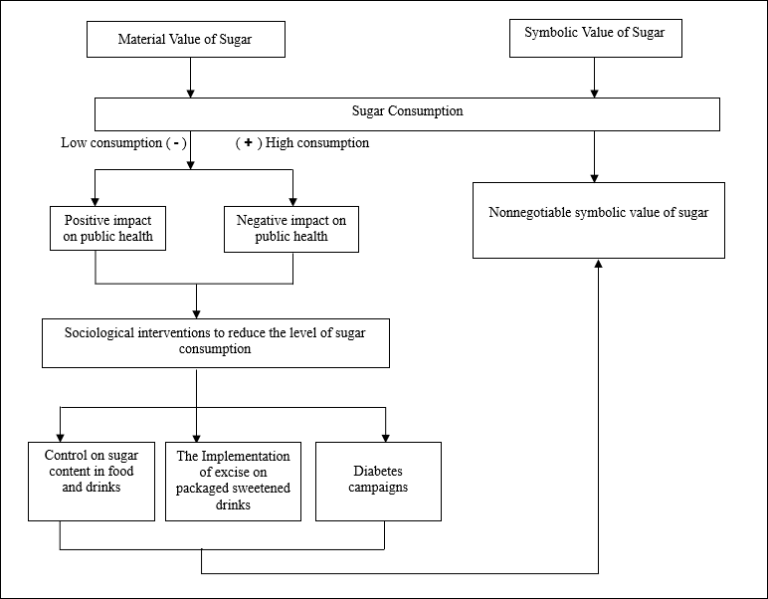
Nonnegotiable symbolic value framework.

Second, the symbolic value of sugar outweighs its material value, leading to its distinctive status as an immutable, culturally or religiously significant food item. This nonnegotiable symbolic value drives high sugar consumption and contributes to negative impacts on public health. Figure 1 summarizes this phenomenon.

#### The Sugar Market: Cognitive Anchoring, Social Positioning, and Standardization

This study analyzes the sugar market through 3 aspects of market valuation by Beckert [5]: cognitive anchoring, social positioning, and standardization. Cognitive anchoring pertains to individual and collective comprehension of markets and products. In the sugar market, it includes knowledge of the market dynamics, pricing mechanisms, and supply-demand factors that shape decisions on purchasing, selling, and investment.

Social positioning concerns the role of social recognition in assigning value to goods. Value is linked to the status the goods confer [19]. For example, wine signifies symbolic prestige beyond material quality. In the sugar market, social positioning involves key stakeholders such as major sugar companies, government entities, and farmer groups in the social and economic hierarchy, which dictates access to resources, information, and influence. Notably, large sugar companies may wield greater influence over pricing compared to small farmers.

Standardization establishes shared criteria for assessing product quality [5]. It is defined as the basis of the physical or material quality of goods, supported by market expansion and digital technology [20] . In the sugar industry, standardization governs how sugar quality and composition are evaluated, shaping consumer perception and global trade valuation.

### Theoretical Framework

Within economic sociology, commodity value derives from both material attributes and embedded symbolic meanings. Beckert [5] argues that markets operate within a social order in which product valuation encompasses material value, referring to the use and tangible benefits of products, and symbolic value, which includes social meanings, status, and identity associated with consumption. Thus, valuation is not merely functional but socially constructed through cultural relations and symbolic meanings.

Symbolic value emerges from interactions among consumers, producers, and prevailing cultural and moral structures. Consumption in this regard is viewed as a symbolic communication system, wherein goods are used to express identity and social position [21]. Meanwhile, the concept of cultural capital [22] suggests that consumption preferences indicate the habitus and class structures that influence consumer behavior. These perspectives demonstrate that symbolic value significantly shapes consumption behavior, often beyond economic rationality.

This study applied the framework of economic sociology by Beckert [5,13] on symbolic value and market valuation, positing that product value emerges from social construction rather than material properties alone. In the context of sugar consumption in Indonesia, this framework elucidates how the emotionally, culturally, and socially embedded nonnegotiable symbolic value of sugar shapes consumption practices. Prior supporting studies on the traditional herbal medicine (jamu) market [20], digital group solidarity [23], and moral embeddedness in the labor market [24] conceptually reinforce the interrelations among social norms, digital interactions, and consumption behavior.

To analyze these dynamics, this study adopted 3 key dimensions of Beckert [5] (standardization, cognitive anchoring, and social positioning) to explain how collective perceptions, social norms, and individual positions within social structures reproduce the symbolic value of sugar. Integrating digital network analyses, specifically TNA and SNA, this study explores how the symbolic value of sugar is constructed, maintained, and reinforced within online public spaces and contemporary digital culture.

## Methods

### Research Methodology

This study used a mixed methods approach with a qualitative digital orientation, integrating TNA and SNA to examine nonnegotiable symbolic value and uncontrollable sugar consumption in Indonesia. This approach explored how symbolic value, public communication, and consumption practices are shaped through digital interactions. Purposive sampling was guided by 3 theoretical dimensions of Beckert [5] (standardization, cognitive anchoring, and social positioning) to capture relevant social contexts and public narratives. The analysis drew from both primary and secondary data. Primary data included web-scraped digital texts and regulatory documents, while secondary data encompassed market observations and interviews with 3 key informants. Data processing and interpretation combined the quantitative rigor of network analysis with qualitative interpretive depth, enabling a comprehensive understanding of the complexity of symbolic value and sugar consumption practices in Indonesia. Validation was achieved through source and method triangulation, member checking, and peer debriefing, ensuring consistency and reliability.

### Data Collection

Data collection began with identifying keywords related to the material and symbolic values of sugar (Table 1), followed by web scraping using NCapture (Lumivero). Primary data were collected from online news articles, YouTube videos uploaded between January 2018 and July 2022 (see Multimedia Appendix 1), and government policy documents. They were cleaned, coded, and categorized in NVivo (Lumivero) based on material value, symbolic value, and social positioning. They were then processed through stop-word filtering in WORDij (Amsterdam School of Communication Research) and imported into Gephi (Gephi Consortium) to generate TNA visualizations of the material and symbolic values of sugar. Concurrently, the SNA visualization mapped dominant actor networks regulating sugar levels in packaged foods and beverages.

**Table 1.** Keywords for web scraping from online media articles.

Criteria	Keywords
Standardization	Standardized sugar in food and beverages, sugar consumption policy standardization, SNI^a^ for sugar, and sweetness level.
Cognitive anchoring	Sugar for diet, low-calorie sugar, experiences with sugar and artificial sweeteners, experiences with sugar and without sugar, reasons for using sugar in foods and beverages, sugar content, reasons for having a fondness for sweet foods, and the symbolic meaning of sugar.
Social positioning	The history and culture of sugar consumption, the royal symbolism of sugar, and the political economy of sugar.

a SNI: Indonesian National Standard.

The material value of sugar, analyzed through standardization, was drawn from 13 online articles and 3 regulations: Permenkes No. 30/2013 on daily sugar intake limits and nutritional information labeling for packaged products, Permendag No. 14/2020 on sugar import provisions, and the Regulation of the National Agency of Drug and Food Control (BPOM) No. 4/2014 on artificial sweetener limits in foods and beverages. The symbolic value of sugar was explored through 106 online articles, 76 on cognitive anchoring and 30 on social positioning, and 7 YouTube seminar transcripts.

Secondary data were obtained from market observations examining labeling compliance in packaged foods and beverages. In addition, online interviews were held between January 2023 and March 2023 with 3 purposively selected key informants: a senior researcher from the National Research and Innovation Agency, a research project officer from the Center for Indonesia’s Strategic Development Initiatives, and a clinical physician who is also a person with diabetes. Discussions addressed the effectiveness of the sugar-sweetened beverage (SSB) excise policy, the implementation of Permenkes No. 30 of 2013 on sugar, salt, and fat (SSF) labeling, consumer literacy, and responses of the food and beverage industry to existing regulations. Informants also highlighted the contribution of SSB to the rising NCD prevalence in Indonesia, assessed current regulatory effectiveness, described market conditions and consumer preferences for sweetness, and suggested effective intervention strategies such as public education, product reformulation, and cross-sectoral policy approaches.

**Figure 2.**
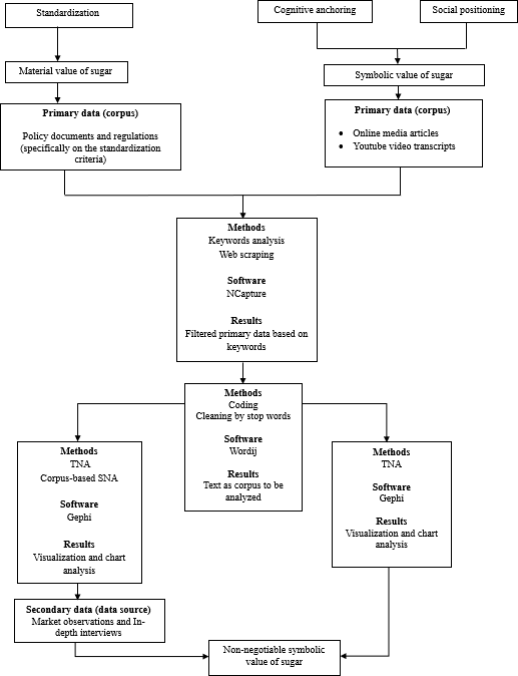
Methodology framework. SNA: social network analysis; TNA: textual network analysis.

These methods enabled the study to map how the nonnegotiable symbolic value of sugar is reproduced in both digital discourse and everyday practices. These findings elucidate its role in sustaining sugar-based dietary habits in Indonesia and inform more comprehensive sociological interventions to control sugar consumption by considering cultural and symbolic dimensions. The methodology framework is presented in Figure 2.

### Ethical Considerations

The study was reviewed and approved by the research ethics committee of the Faculty of Social and Political Sciences, Universitas Indonesia (reference: KET-12/UN2.F9.KEP/PPM.00.02/2025; certificate: SER-12/UN2.F9.KEP/PPM.00.02/2025). The research used secondary and observational data and did not involve direct interaction or intervention with human participants. Therefore, the requirement for informed consent was waived by the ethics committee. Participants did not receive any financial or material compensation. All data were analyzed in an aggregated and anonymized form to ensure confidentiality and prevent individual identification.

## Results

### TNA and SNA Visualizations of the Material Value of Sugar and its Negative Impacts on Public Health

#### TNA of Material Valuation Based on Standardization

**Figure 3.**
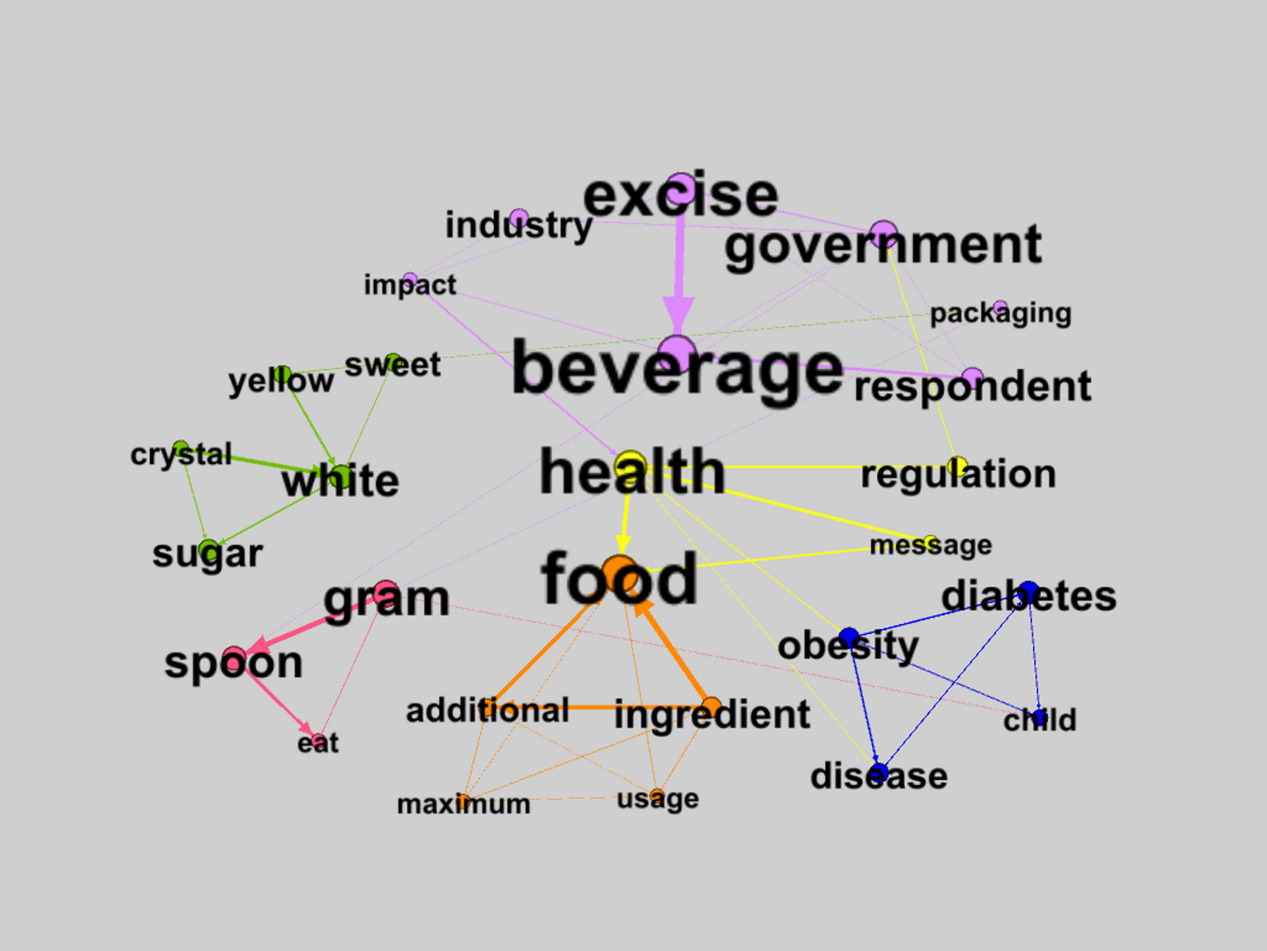
Textual network analysis (TNA) visualization of sugar based on standardization.

The TNA visualization of the sugar and artificial sweetener market, focusing on standardization, reveals 27 nodes and 58 edges with 6 clusters (Figure 3). These clusters include: purple (38/626, 6.07%), green (33/626, 5.27%), yellow (26/626, 4.15%), orange (21/626, 3.35%), blue (20/626, 3.19%), and pink (17/626, 2.72%).

Figure 3 illustrates the structure of public discourse on sugar, food, beverages, and health through several color-coded thematic clusters. The purple cluster centers on beverages and their association with “excise,” “government,” “industry,” “impact,” and “packaging,” framing sweetened beverages as objects of fiscal policy and industrial regulation. The green cluster groups “sugar,” “white,” “crystal,” “sweet,” and “yellow,” reflecting the physical attributes and sensory perceptions of sugar as a consumer product.

**Figure 4.**
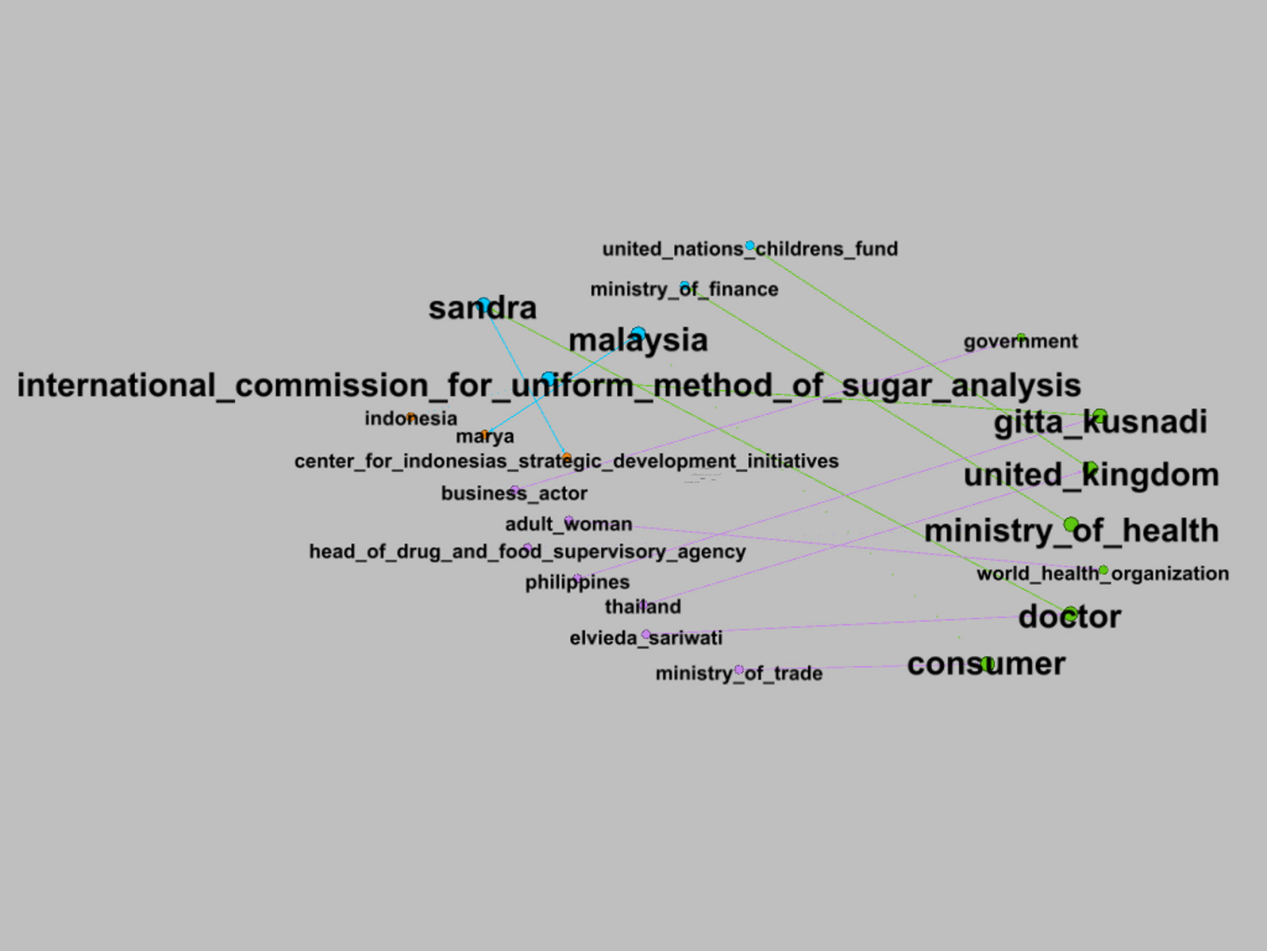
The social network analysis (SNA) visualization of sugar based on standardization.

Figure 4 presents an SNA visualization of sugar-related discourse based on standardization, highlighting key regulatory and industry actors. The yellow cluster comprises “health,” “regulation,” and “message,” highlighting the role of public health as an intermediary between food and policy as well as a channel for communicating regulatory messages. The orange cluster focuses on “food,” “ingredient,” “additional,” “maximum,” and “usage,” depicting technical discourses on food composition and limits on added sugar. The blue cluster connects “diabetes,” “obesity,” “disease,” and “child,” representing narratives of health risks and NDCs associated with sugar consumption, including among children. Meanwhile, the pink cluster links “gram,” “spoon,” and “eat,” indicating measurement and everyday practices of consuming sugar. Overall, this mapping demonstrates that discourse on sugar is segmented into policy, public health, technical food considerations, disease risk, product perception, and consumption practices, without implying a direct causal relationship with individual behavior.

#### SNA of Influential Actors in the Policy on the Sweetness Levels in Packaged Foods and Beverages

The corpus-based SNA visualization focused on standardization uncovers dominant actors in the policy on the sweetness levels in packaged foods and beverages. This visualization comprises 29 nodes and 15 edges, with 4 clusters of actors in reference to eigenvector centrality.

Eigenvector centrality analysis further quantified the relative influence of these clusters (Table 2). The most dominant clusters (4/29, 13.79%) consist primarily of governmental and international health actors such as the Ministry of Health, BPOM, WHO, and ICUMSA, indicating their pivotal role in shaping narratives on sugar regulation and public health. The purple cluster holds the most substantial influence in formulating the sugar content policies for packaged foods and beverages, demonstrating strong interconnections among key stakeholders, including the Ministry of Health, the Ministry of Trade, and BPOM. In contrast, the public and business sectors are expected to exert greater influence following the implementation of these policies.

**Table 2.** Value of eigenvector centrality.

Cluster color	Eigenvector centrality	Value, n/N (%)
Purple	Elvieda Sariwati, MD, adult women, Ministry of Trade, Head of BPOM^a^, Philippines, Thailand, and business actors	4/29 (13.79)
Light green	Ministry of Health, consumers, doctors, Gitta Kusnadi, United Kingdom, WHO^b^, the government	4/29 (13.79)
Blue	ICUMSA^c^, UNICEF^d^, Malaysia, Ministry of Finance, Sandra	4/29 (13.79)
Orange	Marya, CISDI^e^, Indonesia	3/29 (10.34)

a BPOM: National Agency of Drug and Food Control.

b WHO: World Health Organization.

c ICUMSA: International Commission for Uniform Methods of Sugar Analysis.

d UNICEF: United Nations Children's Fund.

e CISDI: Center for Indonesia’s Strategic Development Initiatives.

### TNA Visualizations of the Nonnegotiable Symbolic Value of Sugar in Processed Foods and Beverages Containing Refined Sugar

#### TNA of Symbolic Valuation Based on Cognitive Anchoring

The TNA visualization of the sugar market based on cognitive anchoring reveals 56 nodes and 430 edges with 5 clusters. These clusters include: purple (171/2071, 8.26%), green (148/2071, 7.15%), blue (121/2071, 5.84%), yellow (95/2071, 4.59%), and gray (49/2071, 2.37%).

**Figure 5.**
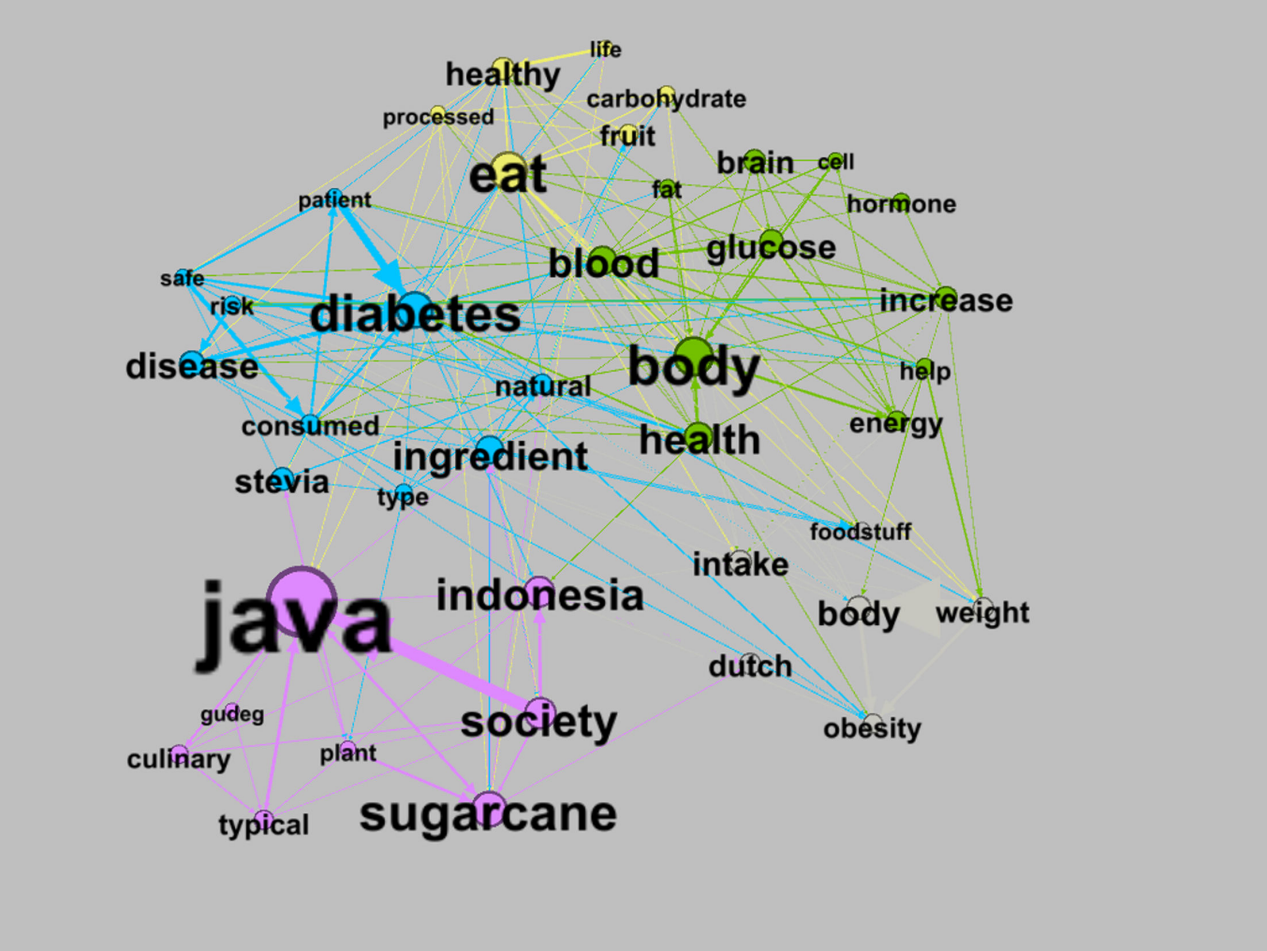
Textual network analysis (TNA) visualization of sugar based on cognitive anchoring.

Figure 5 presents a TNA visualization mapping semantic linkages within discourses on sugar consumption in Indonesia. The word network reveals overlapping thematic clusters, illustrating sugar as not only a nutrient but also a health, cultural, and social issue. The purple cluster (“Java,” “sugarcane,” “society,” and “Indonesia”) represents the sociocultural dimensions of sugar consumption, linking geographical context, sugarcane production, culinary traditions, and the role of sugar in social life and local identity. The green cluster (“body,” “glucose,” “blood,” “health,” “energi,” and “hormone”) reflects the understanding of sugar as part of biological and metabolic processes, with implications for health and body weight. The blue cluster (“diabetes,” “disease,” “risk,” and “patient”) captures medical and public health discourses emphasizing the risks of sugar consumption, particularly diabetes, and preventive approaches grounded in health and safety.

The yellow cluster (“eat,” “healthy,” “carbohydrate,” “fruit,” and “fat”) relates to everyday eating practices, food choices, and healthy lifestyle narratives that connect nutritional knowledge with consumption behavior. The gray cluster (“intake,” “foodstuff,” “weight,” “obesity,” and “body”) reflects health-oriented rationalities and dietary intake control, focusing on measurable impacts on nutritional status and body weight. Overall, the interconnected clusters suggest that sugar consumption is constructed through interactions between material values (eg, “health,” “the body,” and “disease”) and symbolic values rooted in the social and cultural context of Indonesia.

#### TNA of Symbolic Valuation Based on Social Positioning

**Figure 6.**
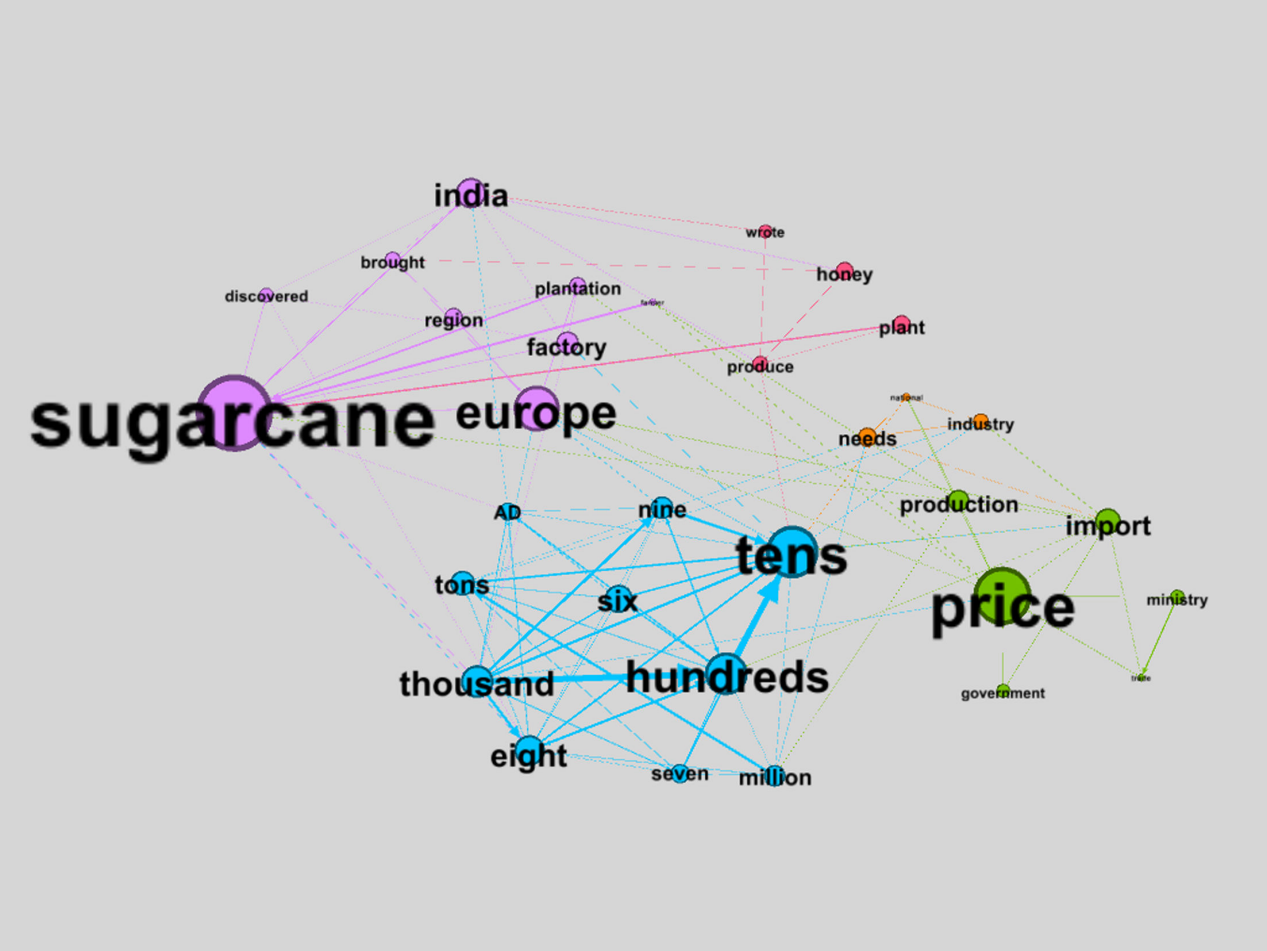
Textual network analysis (TNA) visualization of sugar based on social positioning.

The TNA visualization of the sugar and artificial sweetener market based on social positioning displays 33 nodes and 136 edges with 5 clusters. These clusters include: purple (83/752, 11.04%), green (48/752, 6.38%), blue (30/752, 3.99%), orange (26/752, 3.46%), and pink (23/752, 3.06%). The visualization is presented in Figure 6.

Figure 6 presents a TNA visualization of the discourse structure surrounding sugarcane and the sugar market through interconnected thematic clusters. The purple cluster centers on “sugarcane,” “Europe,” “India,” “plantation,” and “factory,” representing the historical, geographical, and production bases of sugarcane, including colonial diffusion pathways and early production systems based on plantations and factories. The green cluster (“price,” “import,” “government,” and “ministry”) reflects sugar market mechanisms and regulatory frameworks, emphasizing pricing, import policies, and the role of the state and ministries in sugar market regulation and stabilization. The blue cluster is dominated by numerical terms (“tens,” “hundreds,” “thousand,” and “million”), demonstrating quantification, scale, and volume, indicating how sugar-related discourse is frequently framed through measurements of production, distribution, and consumption.

The orange cluster, which includes “industry,” “needs,” and “national,” frames sugar as a strategic commodity for meeting national needs, highlighting the role of industry in ensuring supply and production sustainability. This cluster situates sugar within a macroeconomic and food security framework, in which national consumption requirements underpin policy decisions and industrial activities. The pink cluster (“plant,” “produce,” “honey,” and “wrote”) represents discourses on sweeteners in natural and preindustrial contexts, reflecting early practices and understanding of sweetener sources from plants and honey and their documentation in historical narratives. Overall, the interconnections among clusters indicate that discourse on sugarcane and sugar is constructed through interactions among historical–productive dimensions, economic quantification, market mechanisms, and industrial and policy logics.

## Discussion

### Alternative Sociological Interventions to Reduce Sugar Consumption While Addressing its Nonnegotiable Symbolic Value to Improve Public Health

#### The Implementation of Permenkes No. 30 of 2013

Permenkes No. 30 of 2013, mandating the labeling of SSF content and health warnings on processed and ready-to-eat foods, represents a key strategy for NCD risk control. However, TNA focused on standardization (Figure 3) demonstrates that producers largely ignore the recommended daily sugar intake (maximum 50 g or 4 teaspoons). This implies weak regulatory enforcement and low producer awareness of the right of consumers to transparent information.

**Figure 7.**
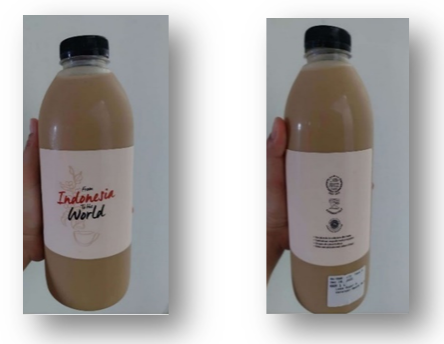
Packaged beverage produced in Indonesia with noncompliant labeling.

Market observations indicate widespread noncompliance among Indonesian food and beverage producers, reflecting weak accountability in protecting consumer health. The absence of restrictions on added or artificial sweeteners further worsens this issue. As presented in Figure 7, many packaged beverages lack clear sugar content labeling, highlighting the urgent need for stricter sanctions.

**Figure 8.**
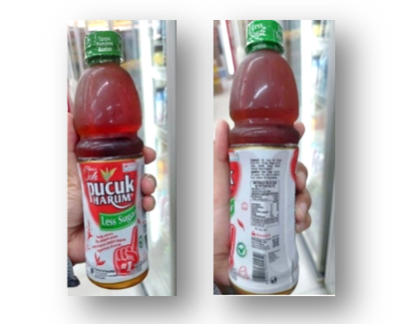
A packaged beverage produced in Indonesia with incomplete labeling.

Several domestic producers partially comply with Permenkes No. 30/2013 by providing basic health information. Figure 8 presents an example of a locally manufactured beverage that includes sugar content, health information, and producer details on its label. However, such instances remain limited, and the quality and completeness of labeling information vary widely across brands.

**Figure 9.**
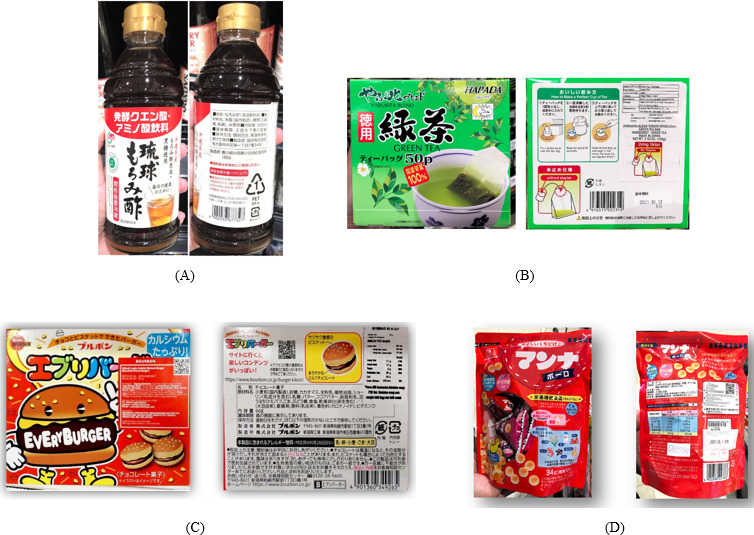
Packaged foods and beverages produced in Japan, including (A) vinegar-based beverage, (B) green tea bags, (C) chocolate biscuit, and (D) biscuit snack.

In contrast, Japanese food and beverage products consistently feature comprehensive labeling, including sugar content, manufacturer details, website, and 2D barcode (Figure 9), enabling consumers to make informed choices. This comparison reveals that Indonesia lags behind Japan in labeling transparency and consumer protection. Strengthening Permenkes No. 30/2013 thus requires not only administrative enforcement but also sociocultural alignment with Indonesian consumption behavior.

The TNA and SNA findings indicate that sugar consumption in Indonesia is shaped by the interrelation between material factors and symbolic meanings embedded in social and cultural contexts. Sugar is perceived not only as a food ingredient, but also as a symbol of pleasure, togetherness, and well-being that is continuously reproduced through public discourse. Within the market valuation framework of Beckert [5], these findings reflect the interplay between material and symbolic values in market practices, where government standardization grounded in health rationality often confronts cognitive associations of “sweetness” with affection, happiness, and social comfort.

Rather than establishing direct causal links to consumption behavior, TNA elucidates the cognitive and cultural context that sustains sugar consumption. The SNA findings complement this by highlighting the role of key actors within regulatory and market networks in maintaining and reinforcing these symbolic meanings. Consequently, policy approaches that rely solely on text-based nutritional labeling tend to be less effective, as they conflict with the deeply institutionalized symbolic understanding of sugar. Front-of-pack color-coded labeling may therefore serve as a symbolic intervention with the potential to challenge these meanings, while bridging public health policy with the social and cultural structures underlying excessive sugar consumption.

#### Revision of Permenkes No. 30/2013

The mandate under Permenkes No. 30 of 2013 to disclose SSF content and health warnings on processed and ready-to-eat foods has not effectively changed public consumption patterns. Interviews with 3 key informants from the National Research and Innovation Agency of Indonesia, Center for Indonesia’s Strategic Development Initiatives, and the medical sector identified 3 main barriers: low nutrition literacy, noncommunicative label design, and weak regulatory enforcement.

An informant from the Public Health and Nutrition Research Center of National Research and Innovation Agency of Indonesia identified low nutrition literacy as the primary barrier. Although nutritional labeling meets global standards, public awareness remains limited. “Many people do not understand or pay attention to details. Even expiration dates are often ignored.” The informant suggested a simplified color-coded system (green, yellow, and red) to communicate product risks more clearly.

Echoing this, an informant from Center for Indonesia’s Strategic Development Initiatives noted that narrative text-based labels are ineffective in a visually driven digital era. Consumers respond more to visual promotions than textual warnings. “There are no regulations on playful characters for children, leaving gaps for improvisation. Health warnings remain non–eye-catching.” The informant further emphasized the need for digitalized monitoring and cross-sector collaboration among the Ministry of Health, BPOM, and the Ministry of Industry to improve compliance.

A medical practitioner and diabetes survivor added that most consumers do not understand or ignore nutritional information. “If people never read the information, the regulation is useless.” This underscores the gap between material value (regulations and factual data) and symbolic value (social meaning attached to sugar), as described by Beckert [5]. In this context, nutritional information lacks sufficient social impact to alter the symbolic perception of sugar as a source of pleasure and energy.

The implementation of article 4, paragraph (2), which warns that “consuming more than 50 g of sugar per day increases the risk of hypertension, stroke, diabetes, and heart attack,” also remains ineffective. Many “healthy” products, such as yogurt, instant oatmeal, and low-fat salad dressing, contain 12 to 26 g of sugar per serving, reinforcing sweetness as a symbol of pleasure and premium value rather than a health risk.

These findings highlight the need to revise Permenkes No. 30 of 2013 to improve both the content and delivery of health warnings. A color-coded system, green for below the daily limit, yellow for 50%, and red for more than 60% to 75%, can enable faster risk interpretation and encourage producer compliance. Beyond administrative compliance, labeling should function as a social intervention to shift the symbolic meaning of sugar from pleasure toward health awareness.

This approach should be integrated with public education through social media, schools, and primary healthcare facilities. Incorporating standardization, cognitive anchoring, and social positioning dimensions from the theory of Beckert [5], the policy can serve as a multidimensional intervention uniting regulatory measures, social literacy, and symbolic transformation to promote healthier consumption behavior.

#### An Ideal Example of Sweetness Level Labels and Health Warnings on Processed Foods

Referring to the guidelines by BPOM in 2020, processed food labels must include: (1) product and trade names, (2) ingredient list, (3) net weight or volume, (4) producer or importer details, (5) halal certification as mandated, (6) production date and code, (7) expiry date, (8) distribution permit number or marketing authorization number, (9) ingredient origin, and (10) color code (green, yellow, and red), as proposed in this study.

Labels should also encompass nutritional values, a 2D barcode, and additional legally mandated information. Products containing artificial sweeteners must include health warnings such as “Intended for diabetics or those seeking low-calorie options. Not suitable for children under five or pregnant/lactating women. Overconsumption may cause a laxative effect.” [25].

Since the mid-2000s, front-of-pack nutritional labeling has been applied in the United Kingdom. In October 2012, the UK government recommended a traffic-light system for front-of-pack, which is now widely adopted by producers. This system communicates total fat, saturated fat, sugar, and salt content: green for low, yellow for moderate, and red for high levels [15].

#### Controlling Sugar Consumption Through the Visualization of Cognitive Anchoring

TNA focusing on cognitive anchoring (Figure 5) reveals strong interactions between the symbolic and material values of sugar across narrative clusters. The purple cluster highlights cultural reasons behind Javanese preferences for sweet foods, framing sugar as a symbol of intimacy, happiness, and prosperity. Within the framework proposed by Beckert [5], this indicates how symbolic value is deeply embedded in cognitive anchoring, making sugar consumption a socially inherited habitus.

Conversely, the gray cluster emphasizes narratives on health risks, such as obesity and diabetes, exposing tension between the material value of sugar as energy and awareness of its health impacts. The blue cluster presents solutions, advocating natural sweeteners such as stevia to preserve the symbolic meaning of sweetness while promoting health.

Indonesian consumers exhibit a strong preference for high-sugar beverages such as packaged tea, with a 250 ml serving containing approximately 21 g of sugar (pink cluster). This suggests that processed sugar carries greater symbolic value than raw sugar. Sweet beverages are frequently associated with refreshment, social bonding, and modern lifestyle, forms of symbolic positioning consistent with Beckert [5]. The longstanding fondness for sweet foods in Java and Yogyakarta, established in childhood, reflects a culturally stable sugar-based habitus.

These findings align with international studies demonstrating that sugar consumption is influenced not solely by biological needs but also by social, cultural, and emotional factors. Sugar has been linked to social rituals such as communal meals and celebrations [26], and it carries a dual meaning, representing both pleasure and a health risk in France and Denmark [27]. In Indonesia, sugar also signifies luxury, happiness, and social status. Globalization has been associated with increasing sweet beverage consumption among adolescents, while family influence and digital education shape sugar consumption among children [28].

Research also underscores the symbolic dimension of sugar in public policy. Marketing strategies leveraging social symbolism significantly affect consumer choices toward low-sugar products, indicating that consumption decisions are often driven by social meaning rather than rational evaluation [29]. Health perceptions, social norms, and public policies such as nutrition labeling play critical roles in reducing sugar intake. Historically, sugar served as a marker of status, power, and social identity from colonial to industrial times [30].

Therefore, controlling sugar consumption in Indonesia requires more than regulatory measures or health campaigns. Effective interventions should integrate symbolic and cultural understanding with the dimensions of standardization (regulation), cognitive anchoring (cultural internalization), and social positioning (actor and market structures) by Beckert [5] to create sustainable social change.

#### Controlling Sugar Consumption Through the Visualization of Social Positioning

The visualization of social positioning in controlling sugar consumption reveals the dominance of the purple cluster, tracing the historical and social evolution of sugar from its early production in Indian sugarcane plantations to its status as a luxury commodity among European elites. Historically, the material and symbolic values of sugar were confined to privileged classes, positioning it as a symbol of luxury, status, and power [5]. This exclusivity established sugar not merely as an economic commodity but as a marker of class distinction.

In modern society, this symbolic legacy endures in a transformed manner. The rising demand for sugar in the food and beverage industry reflects its evolution from a luxury item to a mass symbol of pleasure and modern lifestyle. Despite broader economic accessibility, sugar continues to represent comfort, celebration, and social bonding that continue to drive overconsumption, particularly in urban settings. Consistent with the framework of Beckert [5], social positioning legitimizes the allure of sugar beyond its utilitarian function.

International trade dynamics further highlight the dual nature of sugar as a material necessity and a symbolic commodity. Global expansion and economic centrality demonstrate the persistence of structural power relations, where corporate actors and trade systems not only regulate sugar availability but also shape its social and cultural meanings. Consequently, controlling sugar consumption requires more than economic and regulatory measures. It demands sociological interventions to confront the deeply embedded symbolic value of sugar in everyday life.

#### Sociological Intervention in Sugar Market Valuation Through the Implementation of the Excise Policy on Packaged Sweetened Foods and Beverages

The rising consumption of SSBs is influenced by multiple factors, including their relatively low prices and widespread availability, highlighting the need for sociological interventions to reduce intake, particularly among children and youth [2]. Excise taxes have proven effective in reducing consumption by increasing retail prices. Since 2015, the United States has enforced excise taxes on sugary beverages, causing a 21% decline in consumption. The “Soft Beverages Levy” introduced in the United Kingdom in 2018 reduced sugar intake by 10%, while the excise tax policy applied in Mexico in 2014 achieved a 6%‐8% decrease. Similarly, South Africa adopted the “Health Promotion Levy” in 2018 and observed a considerable 29% reduction in sugar consumption [31].

#### Sociological Intervention in Sugar Market Valuation Through Diabetes Awareness Campaigns

The Health Minister of Indonesia emphasizes proactive strategies to prevent and control diabetes. These include regular risk monitoring and lifestyle modification, particularly in high-risk communities. The government advances this agenda through community empowerment programs under Integrated Development Posts for NCDs.

The Ministry of Health also promotes CERDIK, an acronym for check health regularly, avoid smoking, exercise regularly, eat a healthy diet, rest adequately, and keep balance between body and mind [32]. Effective diabetes awareness campaigns further necessitate community involvement, using testimonials and success stories to motivate healthier behaviors.

#### Dealing With the Nonnegotiable Symbolic Value

This economic sociology study using the framework of Beckert [5] offers key insights into sociological interventions. First, it reveals that consumer behavior is shaped by both the symbolic and material values of a commodity. The nonnegotiable symbolic value in sugar consumption is identified, in which meanings attached to sugar extend beyond rational economic considerations. This enriches the concept of value construction of Beckert [5] that symbolic meanings can persist even when confronted with health risks and economic rationality.

Second, the nonnegotiable symbolic value of sugar manifests through its association with emotional satisfaction, happiness, and social bonding. Sweet foods hold cultural significance in traditions and celebrations, symbolizing generosity, joy, and prosperity, elevating sugar from a mere commodity to a social symbol. Consequently, reducing sugar consumption requires sociological strategies that address its symbolic dimension alongside economic and health-based approaches. Integrating 3 analytical dimensions (ie, standardization, cognitive anchoring, and social positioning) of Beckert [5] clarifies the persistence of sugar consumption in Indonesia.

First, standardization is reflected in state regulations, such as Permenkes No. 30 of 2013, which sets daily sugar intake limits but suffers from weak enforcement and low public awareness. Second, cognitive anchoring is evident in cultural preferences for sweet foods and beverages, reinforced by digital advertising and everyday practices that normalize sugar as a symbol of comfort, happiness, and social acceptance. Third, social positioning involves dominant actors, including major sugar corporations, government agencies, and consumer groups, who shape policy direction, market access, and the symbolic legitimacy of sugar products.

Linking these 3 dimensions to empirical findings demonstrates that high sugar consumption in Indonesia is not merely economic but deeply rooted in social and cultural meanings. This study extends the theoretical framework of Beckert [5] by incorporating the cultural context of Indonesia, digital marketing narratives, and the paradox between high health-risk awareness and persistent overconsumption. It thus reinforces the theoretical relevance of the market valuation model proposed by Beckert in explaining the persistence and complexity of contemporary sugar consumption patterns.

### Critical Discussion: Extending the Symbolic Value Theory in the Digital Era

The symbolic value theory by Beckert [5,15] interprets commodity consumption through social status, identity, and imagined futures. However, its application to sugar consumption in Indonesia remains limited. This study extends the framework by introducing 3 dimensions capturing contemporary social, cultural, and economic shifts.

First, the local cultural dimension. In Indonesia, sugar symbolizes togetherness and tradition beyond social status. Sweet tea and snacks are deemed integral to social interactions in West Java [33]. Unlike the distinction theory [22], sugar consumption is more inclusive and collective, deeply rooted in everyday communal practices.

Second, the digital narrative dimension. Digital promotion normalizes sugary beverage consumption, particularly among youth. Indonesian food and beverage companies are reported to use social media hashtags, promotional characters, and interactive games to target children and adolescents [34]. Similarly, brands such as Coca-Cola frame sugary beverages with happiness, togetherness, and active lifestyles [3]. Thus, the symbolic value of sugar now arises from interactive, cross-cultural, and emotionally charged digital narratives, not merely market mechanisms.

Third, the public health paradox dimension. Despite awareness of diabetes risks, sugar consumption remains high due to cravings, social norms, and digital marketing [33,34]. As observed in France and Denmark, sweetness functions as a socially accepted “guilty pleasure” [27].

Recent reports [17,35] confirm these trends among Generation Z, linking growing diabetes risks to heavy consumption of “Instagrammable” sugary beverages promoted as modern lifestyle symbols. Consequently, the theory of Beckert [5] requires expansion to encompass: (1) digitalization, where social media algorithms and visual content shape perceptions of value and pleasure; (2) globalization, where consumption narratives adapt to global trends; and (3) public health paradox, where heightened health awareness enhances rather than reduces the symbolic appeal of indulgence.

By integrating these dimensions, this study introduces the conceptual model of digital-symbolic value of consumption, where symbolic meanings emerge from interactions among digital capitalism, global aspirations, and youth self-expression. In this concept, sugar consumption in Indonesia transcends social status or collective culture, becoming part of digital identity and global lifestyle. Accordingly, this study advances economic sociology by highlighting how digitalization, globalization, and health ambivalence redefine the symbolic meanings of commodities in the modern era.

### Limitations

The main limitation of qualitative digital research lies in sample determination. Data from online news media and YouTube are difficult to apply due to their uncertain nature. Although digital research may lack repeatability, it remains accountable and fulfills the criterion of recoverability with regard to data collected within a specific time period.

### Conclusions

#### Demand-Side Perspective on Sugar Consumption and Regulatory Compliance

This study contributes to the demand side of economic sociology concerning the sugar market. It explores strategies to address or mitigate the sugar-driven food habits and habitus from the perspective of consumer behavior. Simultaneously, it examines producer compliance with regulations aimed at controlling sweetness levels in foods and beverages, thus supporting efforts to reduce sugar consumption and the prevalence of NDCs.

#### An Illustration of the Material Value of Sugar Which Has a Negative Impact on Public Health as Shown by the TNA Visualization

The TNA visualization of material value based on standardization (Figure 3) highlights that the widespread availability of affordable sweetened foods and beverages has increased the prevalence of NCDs, particularly diabetes. Measures to control sweetness levels are thus crucial to protect public health. Implementing excise taxes on SSB will incentivize producers to reformulate products with greater consideration for consumer health.

#### An Illustration of the Nonnegotiable Symbolic Value of Sugar in Processed Foods and Beverages Containing Refined Sugar as Shown by the TNA Visualization

The TNA visualization of cognitive anchoring (Figure 5) reveals that while raw sugar has limited symbolic value, its use in processed foods and beverages elevates its sociocultural significance, shaping consumer behavior. Meanwhile, the TNA visualization of social positioning discloses that sugar was historically reserved for the upper class (Figure 6), but its accessibility today demonstrates a notable evolution of its symbolic value into a staple commodity accessible to all social groups.

#### Alternative Sociological Interventions to Limit Sugar Consumption and Address Its Nonnegotiable Symbolic Value

Controlling sugar consumption habits and habitus and its embedded symbolic value requires a multifaceted intervention. First, the market valuation of sugar and sweet products should be reshaped through targeted consumer education and campaigns. Second, regulatory measures, including the adjustment of sweetness levels, refinement of Permenkes No. 30/2013, and consistent law enforcement, are essential to ensure compliance. Third, implementing excise taxes on sweetened foods and beverages can discourage overconsumption.

Strict enforcement of labeling regulations mandating the disclosure of SSF content remains crucial, as many packaged products still fail to comply. Addressing policy gaps is necessary to effectively curb sugar consumption and challenge its symbolic value. Moreover, empowering women, particularly housewives, to shape consumer cognition and promote healthy lifestyles highlights the importance of active involvement from the Ministry of Women Empowerment and Child Protection [35].

#### Sociological Intervention in Sugar Market Valuation Through the Imposition of Excise on Packaged Sweetened Beverages

By implementing excise on packaged sweetened beverages, the government can better understand the social ramifications of the policy and the necessary measures to ensure that it benefits public health. Prior to the enforcement, it is imperative to conduct a comprehensive analysis of potential social impacts and establish mechanisms for ongoing monitoring and evaluation. This proactive approach enables the government to promptly identify any emerging issues and make requisite adjustments.

#### Sociological Intervention in Sugar Market Valuation Through Diabetes Awareness Campaigns

Diabetes awareness campaigns aim to change public perceptions of sugar, curb excessive consumption, and foster healthier lifestyles. This involves educating the public about the adverse effects of excessive sugar intake through various channels such as social media, seminars, and educational programs in schools, as well as facilitating access to healthier food and beverage alternatives.

#### The Nonnegotiable Symbolic Value of Sugar in the Community

Sugar holds enduring cultural, economic, and social meanings that resist change. Beyond its material function, sugar embodies symbolic significance that shapes social life. Addressing NCDs thus requires transforming these deeply rooted values which often drive excessive consumption.

This study reveals that sugar consumption in Indonesia cannot be fully explained by conventional theories of symbolic value in economic sociology. While the theory of social distinction [22] links symbolic consumption to hierarchies, among Generation Z in Indonesia, sugar and sweet products signify collectivity and shared experience through celebrations, digital interactions, and everyday rituals. Consequently, symbolic value has shifted from exclusivity to inclusivity, becoming expressions of affection and lifestyle identity.

Existing theories overlook how the normalization of sugar in popular culture fosters collective overconsumption, reinforced by media, advertising, and digital algorithms. In the sugar market of Indonesia, symbolic value arises not only from cultural meanings but also from technological infrastructures that accelerate symbolic diffusion and strengthen emotional attachment. Accordingly, this study extends symbolic consumption theory by emphasizing digital, affective, and habitual dimensions. It proposes the concept of digital-symbolic value of consumption, where symbolic value is mediated by social media, digital marketing algorithms, and globalized consumption trends. Evidence demonstrates that young Indonesians perceive sugar not merely as sustenance but as an expression of digital identity and participation in the global emotional economy [34,36].

## Supplementary material

10.2196/77261 Multimedia Appendix 1 YouTube videos and their transcripts as primary data sources.

## Abbreviations

BPOM: National Agency of Drug and Food Control
CERDIK: check health regularly, avoid smoking, exercise regularly, eat a healthy diet, rest adequately, and keep balance between body and mind
NCD: noncommunicable disease
SNA: social network analysis
SSB: sugar-sweetened beverage
SSF: sugar, salt, and fat
TNA: textual network analysis
WHO: World Health Organization
