# Supplementary material for: Nonnegotiable Symbolic Value and Sugar-Driven Food Habits in Indonesia: Mixed Methods Study Using a Digital Sociological Approach
Source: JMIR Infodemiology. 2026 Feb 27;6:e77261. doi: 10.2196/77261 (PMC12954692; doi:10.2196/77261)
Supplement: Multimedia Appendix 1 [file infodemiology-v6-e77261-s001.docx]

**Link Youtube Videos**

[**https://www.youtube.com/watch?v=OkcZl5CjlYo&list=PLB6fLFcPoOQUXENZN8gT9I26DWC6IRr12&index=1**](https://www.youtube.com/watch?v=OkcZl5CjlYo&list=PLB6fLFcPoOQUXENZN8gT9I26DWC6IRr12&index=1)

[**https://www.youtube.com/watch?v=6PLz4PZQXAw&list=PLB6fLFcPoOQUXENZN8gT9I26DWC6IRr12&index=6**](https://www.youtube.com/watch?v=6PLz4PZQXAw&list=PLB6fLFcPoOQUXENZN8gT9I26DWC6IRr12&index=6)

[**https://www.youtube.com/watch?v=kRX1ymakdz8&list=PLB6fLFcPoOQUXENZN8gT9I26DWC6IRr12&index=7**](https://www.youtube.com/watch?v=kRX1ymakdz8&list=PLB6fLFcPoOQUXENZN8gT9I26DWC6IRr12&index=7)

[**https://www.youtube.com/watch?v=yN3d6R1xpHg**](https://www.youtube.com/watch?v=yN3d6R1xpHg)

[**https://www.youtube.com/watch?v=h2-9Q16RCaA&list=PLB6fLFcPoOQUXENZN8gT9I26DWC6IRr12&index=8**](https://www.youtube.com/watch?v=h2-9Q16RCaA&list=PLB6fLFcPoOQUXENZN8gT9I26DWC6IRr12&index=8)

[**https://www.youtube.com/watch?v=xxgirNj6_g8&list=PLB6fLFcPoOQUXENZN8gT9I26DWC6IRr12&index=9**](https://www.youtube.com/watch?v=xxgirNj6_g8&list=PLB6fLFcPoOQUXENZN8gT9I26DWC6IRr12&index=9)

**Transcripts Youtube Videos**

[**https://docs.google.com/document/d/1lNHx0g-2WBiKhaoTzhbmrdfxN8GiFuzLgxWwWWLJ4do/edit?tab=t.0**](https://docs.google.com/document/d/1lNHx0g-2WBiKhaoTzhbmrdfxN8GiFuzLgxWwWWLJ4do/edit?tab=t.0)

[**https://docs.google.com/document/d/1l7nEoiFLpxC3SqetWYr27NxIQsWZ-sADIkfADxAsLsg/edit?tab=t.0**](https://docs.google.com/document/d/1l7nEoiFLpxC3SqetWYr27NxIQsWZ-sADIkfADxAsLsg/edit?tab=t.0)

[**https://docs.google.com/document/d/1xv6aalvo8tknScLMjnX_q___wmvgbV_e_AnBKZaEKW0/edit?tab=t.0**](https://docs.google.com/document/d/1xv6aalvo8tknScLMjnX_q___wmvgbV_e_AnBKZaEKW0/edit?tab=t.0)

[**https://docs.google.com/document/d/1OdfGRSM8Fj7g1LCVLDNecTjhIE81Jp_k1Ev0zKeMtFc/edit?tab=t.0**](https://docs.google.com/document/d/1OdfGRSM8Fj7g1LCVLDNecTjhIE81Jp_k1Ev0zKeMtFc/edit?tab=t.0)

[**https://docs.google.com/document/d/1l_l9jIcTJZ1GEGqZtVQPaoUBkhJ3B8AXxD8Cu-7okEY/edit?tab=t.0**](https://docs.google.com/document/d/1l_l9jIcTJZ1GEGqZtVQPaoUBkhJ3B8AXxD8Cu-7okEY/edit?tab=t.0)

[**https://docs.google.com/document/d/1qu52c_900MX5akWWkFYQT7HT9aVvWT4VNW4WzIXgfKU/edit?tab=t.0**](https://docs.google.com/document/d/1qu52c_900MX5akWWkFYQT7HT9aVvWT4VNW4WzIXgfKU/edit?tab=t.0)
